# Establishing a standing patient advisory board in family practice research: A qualitative evaluation from patients' and researchers' perspectives

**DOI:** 10.1111/hex.14094

**Published:** 2024-06-16

**Authors:** Jennifer Engler, Fabian Engler, Meike Gerber, Franziska Brosse, Karen Voigt, Karola Mergenthal

**Affiliations:** ^1^ Institute of General Practice Faculty of Medicine at Goethe‐Universität Frankfurt am Main Frankfurt Germany; ^2^ Department of General Practice, Faculty of Medicine Carl Gustav Carus, Medical Clinic III Technische Universität Dresden Dresden Germany; ^3^ Present address: Researcher, Faculty of Medicine Carl Gustav Carus Institute for the History of Medicine, Technische Universität Dresden Fetscherstraße 74 01307 Dresden Germany

**Keywords:** citizen science, family practice, patient empowerment, stakeholder participation

## Abstract

**Introduction:**

Patient and public involvement is vital for high‐quality research. Integrating patients' and providers' perspectives early in research enhances the feasibility and relevance of study results. Within our family practice‐based research network ForN, we established a standing patient advisory board (PAB) to include patients with diverse conditions and experiences. In this paper, we aim to describe the establishment and functioning of a standing PAB in family medicine research from patients' and researchers' perspectives.

**Methods:**

After each PAB meeting, patients and researchers were asked to name anonymously positive and challenging moments in a feedback form with open questions. Researchers were also asked to reflect on how they implemented the discussion content in their research projects. The responses from both groups were transferred to MAXQDA 2018 and analyzed separately using thematic analysis.

**Results:**

We analyzed 40 feedback forms from patients and 14 feedback forms from researchers. The dominant theme in the patients' feedback was ‘exchange’: They positively emphasized the ‘exciting and open discussions’ and the exchange of perspectives with one another and researchers. The clarity of the researchers' presentations and the research topics were appreciated. Researchers also positively highlighted the open atmosphere of the discussions. Presenting their research to the PAB helped most researchers reflect on their research topics from patients' perspectives and implement changes. However, researchers also mentioned several barriers to the implementation of PAB members’ feedback.

**Conclusion:**

The establishment of a standing PAB in family practice research is feasible and productive both from patients' and researchers' perspectives.

**Patient or Public Contribution:**

This study reports the evaluation of the establishment of a standing PAB in family practice research. Board members are involved in the design of studies, the co‐production of interventions and information material, and the interpretation of data.

## 1 INTRODUCTION

Well prepared patient and public involvement (PPI) is an integral part of high‐quality research and an effective tool to prevent so‐called ‘research waste’.^1^, ^2^, ^3^ Integrating patients' and providers' perspectives in research in an early stage fosters the feasibility of research projects. Furthermore, PPI contributes to the development of patient‐relevant care solutions within studies and increases the transferability of study results into primary care. Therefore, the establishment of formats and structures for stakeholder involvement—that is the involvement of family practitioners, health care assistants and patients—is a significant feature in most family practice‐based research networks (FPBRNs) in Germany.^4^, ^5^

This development was boosted in 2020 when the Federal Ministry of Education and Research funded six regional and transregional FPBRNs encompassing 23 academic family medicine departments and a coordination office within the initiative DESAM‐ForNet.^6^ The initiative aims to foster high‐quality research in the outpatient setting by developing sustainable, reliable and scalable research structures that compare to research structures in the inpatient setting.^6^ Over the course of time, FPBRNs will add evidence that reflects family practitioners', medical health assistants' and their patient populations' tasks and needs to the overall body of evidence on prevention, diagnostics and therapies. FPBRNs have started to develop qualification programs for research practices, worked on solutions to gather patient data from research practices, conducted several (clinical) interventional pilot studies within the networks and are in close contact with family practices all over Germany.

To incorporate patients' perspective in research within our FPBRN network Frankfurt am Main (ForN)^7^ we decided to establish a patient advisory board (PAB) as an initial component of our network structures.^5^ Different from approaches of study‐specific PABs, we aimed to establish a standing PAB that is located within our FPBRN and selectively involved within different studies. Furthermore, we aimed to include patients that represent the broad patient population from family practices, that is, from all ages, genders, social backgrounds and with and without preconditions. We chose the term ‘patient advisory board’ and addressed potential members as ‘patients’, because we aimed to focus on their role in family practice, namely patients. With regard to the inclusion criteria, we could also have chosen the term ‘citizens’. The diversity of conditions and experiences of PAB members is another distinct feature compared to other study‐specific PPI approaches in healthcare that often include patients with a similar medical condition.^8^ Subsequently, we aimed to include persons who contribute their individual everyday experiences with healthcare in family practice in contrast to patient representatives from patient organizations with a focus on a specific condition. We did not actively reach out to caregivers, even though some patients may hold a double role.^9^

In this paper, we describe the establishment of a standing PAB within our FPBRN ForN, outline methods and content of the PAB's research involvement and present PAB members' and researchers' perspective on these processes.

## 2 MATERIALS AND METHODS

The reporting in this article follows the GRIPP2 Reporting Guideline.^10^

### 2.1 PPI strategy and level of PPI

We aimed to establish a standing PAB as part of a sustainable and ready‐to‐use research structure and to create a relationship of two‐way learning and mutual trust.^11^ This is to be fostered by a coordinator as a stable contact person that organizes PAB meetings, is responsive to barriers and questions from PAB members and operates as a mediator between the FPBRNs' different study teams and the PAB. The coordinator was trained and experienced in qualitative and participatory methods as well as workshop design and moderation. PAB members are involved via participatory workshop meetings (3–4 per year) or in one‐on‐one consultations, for which they get financially reimbursed. Predominately, the level of involvement is defined as ‘consultation’, that is, ‘asking members of the public for their views and use these views to inform decision making’.^12^

### 2.2 Recruitment

We aimed to include patients that represent the broad patient population from family practices, that is, from all ages, gender, social backgrounds and with and without preconditions. Therefore, we had no inclusion criteria besides the ability to participate and communicate in PAB meetings. Furthermore, to maintain clarity of roles, we decided to exclude persons with a background in health care research.

We used several multimodal recruitment strategies and recruited patients between August 2021 and April 2022. (1) We developed an information flyer with a prestamped response postcard that we handed over to 10 interested research practices for display in their waiting rooms. Furthermore, we asked pharmacies to display our information flyer. (2) We talked to interested family practitioners about the PAB, handed over information material and asked them to approach patients they deemed interested individually. (3) We asked patient participants from a former study, in which an intervention was co‐designed,^13^, ^14^ if they would like to join the PAB. (4) We contacted the coordinator of the standardized patient programme at the Frankfurt University Hospital and asked him to approach standardized patients individually with our PAB information materials. Standardized or simulated patients support medical education by acting like a patient with a certain disease. As they are used to communicating with medical staff, we hoped their barrier to involvement is low, even though they have no formalized medical knowledge and their input is based on personal experiences. (5) We developed a workshop for the citizen sciences programme at Goethe University Frankfurt. The programme's schedule of lectures and workshops open to the public is available in print and online.

When patients contacted us, we asked them for a personal phone call. Within this call, we introduced the FPBRN and the Institute of General Practice to them, elaborated their role and tasks as a PAB member, informed them about planned meeting sequences, provided room for questions and announced the date for the next planned onboarding workshop. After the phone call, we sent a short questionnaire that asked for contact information, gender, age, medical conditions and their preferred format for PAB meetings (digital or face‐to‐face) as well as consent to data processing.

### 2.3 Training

#### 2.3.1 Onboarding workshop

We designed an onboarding workshop that included information on our FPBRN as well as research topics covered at our institute and introduced the stages of a research project together with examples of patient involvement at each stage. After each information input, we planned for a short group discussion so that PAB members could get to know each other, that is, their expectations of the PAB, their experiences with family medicine and which aspects of family medicine research they found most interesting. We made clear that there is no duty to share experiences and that they could select which parts they wanted to share with the group. Furthermore, we asked everyone to grant confidentiality to experiences shared within meetings.

#### 2.3.2 Technical introduction workshop and technical support

Each PAB member was offered a technical introduction workshop in which the functions of the video conference system were practiced. Furthermore, one team member was available during each workshop to solve technical problems with the video conference system via phone.

#### 2.3.3 On‐the‐job training

PAB members were informed about the topic and the attending researchers of each PAB workshop via an invitation email. We aimed to minimize the need to prepare in advance, therefore we designed each meeting in a way that allowed PAB members to participate in a meaningful way without preparation. To achieve this, attending researchers were asked to prepare a methods section that introduced the study design and methods of the study that was discussed in the following workshop as well as basic information on the overall aim of the presented study. This ‘on the job’‐training should step by step enhance PAB members' knowledge of research methods while these methods were always presented in the context of the actual study and the workshop on this study. In this manner, we aimed to combine methodological training with study content and therefore to contextualize the PPI activity within the study setting and vice versa. This approach also facilitated researchers' training in PPI ‘on the job’ by developing PAB workshops together with the PPI coordinator. As we aimed to implement and expand PPI activities within the study teams of the FPBRN, we provided methodological counselling in PPI when necessary. Researchers with little experience in PPI could approach the coordinator with a topic they wished to be reflected from the patients' perspective and the coordinator worked together with the researchers to develop a feasible workshop design by reflecting together on question such as: What is a realistic aim for a 2 h workshop and how much content can be discussed within this time? What is the most important question to be discussed? Which changes to the study are actually possible? Which background information is needed for PAB members to discuss the topic? How is this content best presented and prepared for a nonscientific audience?

#### 2.3.4 Glossary

We started a glossary in the onboarding workshop and asked PAB members to write each unclear term into the chat. A member from the academic team explained the term immediately and the term was inserted into a glossary that was adapted after each meeting, emailed to participants and displayed in the secure PAB section of the FPBRN's website.

### 2.4 Evaluation

The literature on evaluation of PPI is diverse. While some authors claim that we need to focus more strongly on PPI as a social interaction with regard to power relations, ‘space to talk’ and ‘space to change’,^11^, ^15^, ^16^, ^17^ others stress that we need more information on the actual impact of PPI on research, that is, what did really change by involving patients and stakeholders.^18^, ^19^ Most authors emphasize, however, that we need more information and more reporting on PPI activities altogether.^10^, ^16^, ^18^, ^19^, ^20^, ^21^

In our evaluation of the PAB's activities, we addressed both PPI as a social interaction from PAB members' and researchers' perspectives and assessed PPIs' impact from researchers' perspectives.

#### 2.4.1 Evaluation from PAB members' perspectives

After each onboarding workshop and each PPI workshop, we asked PAB members to comment on the workshop via a short online feedback form containing three open questions on process and social interaction: 1—what did you like best today? 2—what did you miss today? 3—is there anything else you want to share with us?

The anonymous written answers were transferred onto an Excel sheet and inserted to MAXQDA 2018. We analyzed answers grouped into feedback to the onboarding workshops and project‐specific PAB meetings. Using thematic analysis,^22^ we used a deductive approach first and grouped data with regard to the three questions in the online feedback form. The data was then coded inductively: Answers were coded multiple times when they included multiple aspects. Finally, the codes were grouped into themes. These themes are presented in the results section with exemplary quotations from PAB members. However, marginal experiences are also mentioned in the results.

#### 2.4.2 Evaluation from researchers' perspectives

To assess the social interaction within the PAB meetings we asked researchers, similar to PAB members, after each PAB meeting, (1) what they liked best today and (2) what they felt was challenging. To assess PPIs impact, we further asked (3) with which aim they had involved the PAB, (4) if they felt this involvement was beneficial for their research and what should be different next time to make it more beneficial, (5) which changes to research were made due to the PAB meeting and (6) whether there was input from the PAB that was not included in the research and why. Written answers were inserted to MAXQDA 2018 and analyzed using thematic analysis. First, we used a deductive approach and grouped data with regard to the 6 questions of the feedback form. The data was then coded inductively: Answers were coded multiple times when they included multiple aspects. Finally, the codes were grouped into themes. These themes are presented in the results section with exemplary quotations from researchers. Marginal experiences are also mentioned in the results.^22^

## 3 RESULTS

### 3.1 PAB members

Today the FPBRNs' PAB has 11 members ranging from 17 to 70 years with and without pre‐existing conditions (see Table 1). Only one patient preferred digital to face‐to‐face meetings at the recruitment stage. Nevertheless, the COVID‐19 pandemic forced us to hold most meetings digitally. No PAB member resigned because of the predominantly digital format.

**Table 1:** Sociodemographic description of PAB members (*N* = 11).

	*n*
Gender	
Female	8
Male	3
Age	
17–40	1
40–60	3
60 or older	6
Missing	1
Pre‐existing condition(s)	
Yes	7
No	4
Meeting preference	
Digital	1
Face‐to‐face	3
No preference	7

Abbreviation: PAB, patient advisory board.

### 3.2 Recruitment strategies

The most successful recruitment strategy for patients to become PAB members was when they were informed about the PAB individually by their family practitioner. No patient was recruited via the display of flyers and information material in family practitioners' waiting rooms only. Two patients contacted us because they were informed about the PAB by a friend: a recruitment strategy we did not plan for in advance (Table 2).

**Table 2:** Success of recruitment strategies.

Recruitment strategy	Recruited PAB members
Display of flyers and information material in family practitioners' waiting rooms and in pharmacies	0
Family practitioners informing patients individually about the PAB	4
Asking former study participants	2
Simulated patient programme of the university hospital	2
Information workshop within universities citizen science programme	1
PAB members informing friends	2

Abbreviation: PAB, patient advisory board.

### 3.3 PPI workshops and PAB activities

From October 2021 to July 2023, we conducted two digital onboarding workshops for training and trained one PAB member individually. We conducted three digital and two in‐person project‐specific workshops in which the PAB gave input on research projects of the FPBRN. At these workshops, the coordinator of the PAB was present together with researchers from the project in question. Three PAB members gave feedback on two lay‐language brochures with project results. We invited the PAB to the ‘Day of Family Medicine’ at our university hospital, and three PAB members joined us for lunch and the keynote lecture on ‘Patient Involvement in Family Medicine Research’. Furthermore, PAB members joined the anniversary celebration of our FPBRN and two of them took part in a plenary discussion on ‘Research in the FPBRN as an interprofessional undertaking’ (Table 3).

**Table 3:** Activities of the PAB.

	Format	Topic	Aim	Methods	Participants
Onboarding WS1 and WS2	Digital	Aims and structure of the FPBRN; patient and public involvement	Informing patients on the FPBRNs’ activities and possibilities of getting involved in research; discussing patients’ areas of interest, getting to know each other	Round of introductions; presentations; group discussions; questions and answers	WS1: 7 PAB members, 2 coordinators WS2: 3 PAB members, 2 coordinators
Project‐specific WS1	Digital	Health promotion and prevention in family practice	Assessing patient perspectives on results from interviews with family practitioners and health care assistants on health promotion in family practice	Input presentation on study results; input presentation on the method of qualitative interviews; presentation of a persona (standardized patient case) that wishes to change his lifestyle; collecting and discussion of motivating and demotivating communication strategies; simultaneous documentation via digital white board	6 PAB members, 1 coordinator, 2 researchers
Project‐specific WS2	Digital	Health care for older patients supported by case managers in care networks	Assessing patient perspectives on results from interviews with older patients on case management	Group discussion on patients’ perspectives on getting older at home; presentation of study results and open discussion	8 PAB members; 1 coordinator, 1 researcher
Project‐specific WS3	Digital	Development of a project proposal for a systematic review on the use of herbal medicine	Selecting indications for the development of the search string	Input presentation on herbal medicine, input presentation on the method of a systematic review; discussion on patients’ individual experiences with herbal medicine; simultaneous documentation via digital white board	7 PAB members, 2 coordinators, 2 researchers
Project‐specific WS4	In‐person	Development of information material for patients that are newly‐diagnosed with hypertension	Assessing user experience, feasibility and comprehensibility of prototype material from patients' perspective for adaptation of the material	Sharing individual associations on health promotion via picture postcards; discussion of information material (postcards on lifestyle changes and secondary disorders) following guiding questions	5 PAB members, 1 coordinator, 2 researchers
Project‐specific WS5	In‐person	Discussion of a patient questionnaire on health apps for patients with depression	Assessing the comprehensibility and relevance of the questionnaire from patients’ perspectives	Sharing experiences with health apps in general; sharing of the pilot questionnaire; open discussion of each item	8 PAB members, 1 coordinator, 3 researchers
Day of family medicine	In‐person	Healthcare (research) in family practice	Fostering exchange between PAB members, family practitioners, health care assistants and researchers	Casual talk at lunch; joint attendance of the keynote lecture	3 PAB members together with 2 coordinators
FPBRN anniversary celebration	In‐person	Achievements and future perspectives of the FPBRN	Fostering exchange between PAB members, family practitioners, health care assistants and researchers	Presentations; plenary discussion, casual talk at coffee breaks and dinner	3 PAB members together with 1 coordinator
Feedback on two lay‐language brochures	Per email	Health promotion in family practice; multimedication in middle‐aged patients	Increasing the comprehensibility of lay‐language brochures on study project results	PAB members were sent a draft via e‐mail including a task description; they sent back a version including comments and suggestions for improvement	6 PAB members (3 for each brochure)

Abbreviation: FPBRN, family practice‐based research network; PAB, patient advisory board; WS, workshop.

### 3.4 Evaluation from PAB members' perspectives

We analyzed 10 feedback forms on two onboarding workshops and 30 feedback forms commenting on five project‐specific workshops.

#### 3.4.1 Onboarding—Intelligible information and congenial atmosphere

Concerning the onboarding workshops, PAB members positively stressed the intelligibility of the information provided. Concerning content, they especially liked the display of PAB members’ roles and tasks and the introduction of the FPBRN. The responsiveness of the researchers who moderated was stressed: ‘It was a very comprehensible and informative orientation meeting. I am very happy to be able to participate. The coordinators chaired the meeting very well and with a lot of empathy’. PAB members liked that ‘everything was explained, in a friendly and patient manner’. PAB members furthermore mentioned the ‘congenial open atmosphere’ and felt that they were a ‘good mixture’.

Two participants wished for more time get to know the other PAB members and a comprehensive introductory round. One member wished to meet in person.

#### 3.4.2 Project‐specific workshops—Exchange of perspectives and exciting topics

In PAB members’ feedback on the benefits of the project‐specific workshops ‘exchange’ was the predominant topic: The PAB members stressed that they liked the exchange of ideas and perspectives with other PAB members, the ‘exciting and open discussions’ and the extra time to get to know each other. Similar to the onboarding workshops, PAB members liked the ‘intelligible presentation’ and ‘graphic explanations’. They also mentioned the content of the five project‐specific meetings positively. They liked the ‘interesting information’ and the ‘exciting, future‐oriented topic’. One PAB member summarized: ‘It was highly informative. I liked the topic, the presentations and the exchange very much’.

Answers on what PAB members felt was lacking were heterogeneous. While most had no wishes, the wish for more time to answer questions and give input was articulated twice. Two persons wished for more information on how the PAB members’ feedback was included into the research projects, and one person wished to get to know how the project overall went on. Furthermore, in‐person meetings were wished for twice and one person wished for materials in advance to prepare for the meetings.

The fourth and fifth meeting finally took place in person. The members present stressed their appreciation of the ‘personal and direct’ in‐person discussions and felt that ‘meeting in‐person helps us to move forward’.

### 3.5 Evaluation from researchers' perspectives

We included 14 feedback sheets on five project‐specific workshops from researchers in the analysis.

Similar to PAB members, researchers very often underlined the open and lively discussions within the PAB: ‘[I liked best] that everyone was involved, experiences were shared openly and a dialogue evolved between board members and researchers’. Mentioned challenges encompassed time management and appropriate communication: one researcher found it hard to interrupt because discussions were so lively and enthusiastic while another one found it challenging to ‘keep the flow of the conversation running’. Furthermore, the preparation of study results for a patient audience was mentioned as a difficult task, while this preparation was also seen as a benefit, because it helped to reflect again on the projects’ most important results, anticipating the patients’ perspective.

All researchers felt the PAB meetings were helpful and productive. Two project‐specific workshops discussed study results with patients. In these cases, concrete changes could not be named while the PAB's input helped researchers in weighing their assumptions and research findings from patients’ perspectives and deciding on future research: ‘The workshop underlined our findings from patients’ perspectives, respectively a certain topic was strengthened that patients felt was especially important’.

In three other workshops, PAB members were involved in studies in progress, that is, the selection of indications for a systematic review proposal, checking a patient questionnaire on comprehensibility and relevance and giving feedback on a prototype of information material on hypertension. In these cases, researchers also felt that the PAB's input was beneficial and improved the research a lot, while it was easier for them to name concrete changes to the study based on PAB members’ input. However, most researchers also highlighted obstacles in transferring the PAB's input into research. For example, one researcher mentioned that it might be challenging to decide which input to prioritize given the diverse and sometimes contradicting perspectives of the PAB members. Furthermore, structural and methodological barriers were mentioned such as using standardized items in a questionnaire that therefore can hardly be changed as well as the limited overall length of the questionnaire: ‘When it comes to validated items for the calculation of an index – there's very little room for adaptions. That's why we cannot implement some of the PAB's recommendation for methodological reasons’. Researchers also named time constraints and deadlines from funding agencies as barriers to fully integrate the PAB members’ feedback. In other cases, the processing of the PAB members’ feedback depended on cooperation partners and was therefore not predominantly in the hands of the attending researchers: ‘Naming concrete changes is difficult, because we do not solely decide about the implementation. Having said this, I believe that the PAB's stressing of personal communication between patients, health care assistants and family practitioners was important for the future course of the project and that the PAB affected this future course’.

## 4 DISCUSSION

PAB members stressed the fruitful and open atmosphere, appreciated the changing topics of each meeting and liked the exchange of ideas and perspectives with one another and the researchers. The building of this relationship succeeded, even though most meetings took place in a digital setting by planning for time to get to know each other and social interaction within each meeting. With the end of pandemic‐related restrictions of social contact, many PAB members strongly appreciated meeting in person. Others pointed out the increasing challenge of combining PAB activities with work duties when travelling to in‐person PAB meetings. In the future, a mix of in‐person and digital meetings seems feasible.

The most successful recruitment strategy was family practitioners inviting patients personally to join the PAB. Other successful recruitment strategies also involved personal interactions, while the sole display of flyers in family practices and pharmacies did not motivate any patients to join the PAB. This stresses the importance of trust and sustainable relationships in PPI, while it also raises the question of representation (see Section 5).

The preparation of research material for workshops with the PAB was seen as demanding by some researchers, while it paid off both for researchers—who reflected on the significance of their research for patients and the public—and for PAB members who appreciated the ‘intelligible presentation’ and ‘graphic explanations’ a lot. While all researchers felt that the PAB meetings played a crucial role in weighing findings and emphasizing certain aspects of their projects, some researchers could not name concrete changes that were based on the PAB meetings. This was partly due to the content of the meetings, that is, discussions of project results, but also to methodological and structural barriers to implementation such as standardized questionnaire items, deadlines from funding agencies or the need to come to terms with cooperating partners. These barriers relate to contemporary research structures that are in many cases highly formalized, competitive, involving multiple players and dependent on project‐based external funding. In these surroundings, the topic of providing ‘space to talk’^11^ but also providing and being transparent with regard to ‘space to change’^11^ is especially important. Researchers must communicate openly on research structures, but also on the choices they make and the reasons for these choices when it comes to actual changes made to research projects based on PPI. This is important to prevent ‘sham participation’^12^, ^23^ and because PAB members stressed the importance of being informed about the impact of their meetings and the progress of the research projects they discussed.

Concerning authorship and acknowledgement of contributions to research,^24^ we initiated a discussion within the PAB on the importance of visibility by providing individual names and the possibility of protection by using a group identity. The PAB decided that they do not want their names to appear on the FPBRN's website or elsewhere. In publications the PABs' contribution is honoured in the acknowledgements. With regard to the current level of involvement that is the PAB's counselling on research projects within single sessions, coauthorship was not feasible so far, but this may change in the future. In case individual members decide to contribute to research‐associated events such as panel discussions, they are represented by name just like all other speakers. The PAB's decision on this topic is a matter of constant reconsideration by members.

The COVID‐19 pandemic and the switch to digital formats might have prevented some patients from joining the meetings that were predominantly digital during the pandemic. At first, we hesitated to start the PAB in an online‐only environment. Because of very positive experiences with digital PPI and encouraging evaluation results from patients in a study on multimedication,^13^ we decided to get started anyway. We implemented the supporting tools used in the study such as technical introduction workshops and technical support throughout the meetings and incorporated extra time for discussions and getting‐to‐know each other.^13^ None of the PAB members dropped out during the pandemic because of the digital format, but some might not have joined at all due to barriers in soft‐ and hardware. On the other hand, we know from other studies as well as feedback from PAB members that digital formats can also reduce barriers, as travel restrictions do not apply and participants can tailor their personal environment to suit their individual needs.^13^, ^25^, ^26^ At the end of the pandemic, most PAB members wished for a meeting in person and felt that ‘meeting in‐person helps us to move forward’. We will focus on the shift from online to in‐person meetings and how this may influence communication dynamics within the PAB.

## 5 LIMITATIONS

Even though we theoretically gave everyone interested and present in a family research practice the chance to join the PAB by displaying flyers in waiting rooms, our recruitment strategies might be selective. This might be especially true as most patients joined by personal invitation through their family practitioners, and we have no information why family practitioners approached which patients. This touches the topic of representation, that is always a matter in PPI, when it comes to a selected group of patients speaking for a larger group.^27^ We aimed to approach PAB members as patient experts on eye level and therefore decided to not collect a lot of private, health‐related data from them. Therefore, we can only draw conclusions on the diversity of the PAB on the basis of age, gender and pre‐existing health condition (yes or no). Even though our PAB does represent a wide range of ages and health conditions, we cannot provide information on demographics like migration status or educational level. Also, our initial recruitment strategy was not based on either of these characteristics, but we aim to consider this in the future. Furthermore, we aim to stress that our PAB consists of persons that contribute their individual everyday experiences with healthcare in family practice, given the fact that we ruled out patient representatives from patient organizations. By doing so, we aimed to prevent a special condition from becoming the focus of our discussions in which the family practice is always at the centre. Nevertheless, this focus on individual experiences also excludes the wide range of background knowledge and accumulated knowledge of different patient experiences that patient representatives may provide.

Finally, the evaluation presented in this article is based on PAB members' and researchers' feedback on a couple of single PAB meetings. Even though we collected feedback data at several points in time, our evaluation data contains no information on PAB members' experiences with the overall PPI process within the FPBRN, i.e. if they had wished for more training, a different level of involvement, or another PPI format different from group workshops. In the future, we plan for an overarching evaluation that shall assess patients' overall experiences with the PAB. There are some standardized tools to assess patients' experiences with PPI as well as frameworks that will inspire our evaluation.^28^, ^29^, ^30^ Nevertheless, we aim to develop a guideline for qualitative interviews that addresses the specific tasks, processes and structures of the FPBRN and the PAB within this network to adjust the PAB and PPI activities accordingly.

Concerning the researchers’ perspectives, our evaluation results are limited as well. First, similar to patients, researchers were surveyed at one point in time only, that is 1–2 weeks after the workshop. Reflections, processes and changes to research that occurred after this period could not be assessed. Second, our evaluation is limited to those researchers within the FPBRN that had direct contact with the PAB within a workshop. Most probably these researchers had a positive mindset and were open towards PPI. An extended evaluation could survey all researchers of the FPBRN and assess their attitudes towards PPI in general as well as their knowledge and perception of the PAB to assess the structural and longitudinal changes that the PAB initiated.^28^, ^29^, ^30^ The evaluation results will then inform future directions of the PAB and of PPI activities within the FPBRN in general.

## 6 CONCLUSION

The establishment of a standing PAB in family practice research is feasible and productive both from patients' and researchers' perspectives. PABs should be considered an integral part of research infrastructure in family practice research and beyond and their establishment should be fostered further.

## AUTHOR CONTRIBUTIONS

**Jennifer Engler**: Conceptualization; investigation; methodology; writing—review and editing; writing—original draft; project administration; formal analysis; resources; supervision; data curation; validation. **Fabian Engler**: Writing—review and editing; data curation; investigation. **Meike Gerber**: Writing—review and editing; investigation; data curation. **Franziska Brosse**: Writing—review and editing. **Karen Voigt**: Writing—review and editing; funding acquisition; supervision; project administration; resources. **Karola Mergenthal**: Supervision; resources; project administration; writing—review and editing; Conceptualization.

## CONFLICT OF INTEREST STATEMENT

The authors declare no conflict of interest.

## ETHICS STATEMENT

We informed the local ethics committee of The University Hospital of Goethe University Frankfurt am Main about our intention to establish a patient advisory board (PAB) and to hold patient and public involvement workshops with PAB members. The ethics committee expressed no concerns and waived a formal approval on the basis of the Medical Association's professional code of conduct in Hesse/Germany (§ 15 BO hess. Ärzte). All PAB members gave written informed consent to the processing of workshop results for academic purposes.

## Data Availability

Evaluation data generated and analyzed for this publication is not publicly available, because qualitative data cannot be anonymized totally due to context and content, and we guaranteed patient advisory board members and researchers not to pass on data to third parties.
